# Serotype distribution and antibiotic resistance of *Streptococcus pneumoniae* isolates from 17 Chinese cities from 2011 to 2016

**DOI:** 10.1186/s12879-017-2880-0

**Published:** 2017-12-29

**Authors:** Chunjiang Zhao, Zongbo Li, Feifei Zhang, Xiaobing Zhang, Ping Ji, Ji Zeng, Bijie Hu, Zhidong Hu, Kang Liao, Hongli Sun, Rong Zhang, Bin Cao, Chao Zhuo, Wei Jia, Yaning Mei, Yunzhuo Chu, Xuesong Xu, Qing Yang, Yan Jin, Quan Fu, Xiuli Xu, Hongling Li, Lijun Wang, Yuxing Ni, Hongjie Liang, Hui Wang

**Affiliations:** 1 Department of Clinical Laboratory, Peking University Peoples Hospital, Beijing, 100044 China; 2 Department of Clinical Laboratory, Southwest Hospital, Chongqing, China; 3 Department of Clinical Laboratory, The First Affiliated Hospital of Xinjiang Medial University, Ürümqi, China; 4 Department of Clinical Laboratory, Huazhong University of Science and Technology Tongji Medical College Affiliated Pu Ai Hospital, Wuhan, China; 5 Department of Clinical Laboratory, Zhongshan Hospital, Fudan University, Shanghai, China; 6 Department of Clinical Laboratory, Tianjin Medical University General Hospital, Tianjin, China; 7 Department of Clinical Laboratory, The First Affiliated Hospital of Sun Yat-sen University, Guangzhou, China; 8 Department of Clinical Laboratory, Peking Union Medical College Hospital, Beijing, China; 9 Department of Clinical Laboratory, The Second Affiliated Hospital of Medical School of Zhejiang University, Hangzhou, China; 10 Department of Clinical Laboratory, China-Japan Friendship Hospital, Beijing, China; 11 Department of Clinical Laboratory, Guangzhou Institute of Respiratory Disease, Guangzhou Medical University, Guangzhou, China; 12 Department of Clinical Laboratory, General Hospital of Ningxia Medical University, Yinchuan, China; 13 Department of Clinical Laboratory, Jiangsu Provincial Peoples Hospital, Nanjing, China; 14 Department of Clinical Laboratory, Shengjing Hospital of China Medical University, Shenyang, China; 15 Department of Clinical Laboratory, China-Japan Union Hospital of Jilin University, Changchun, China; 16 Department of Clinical Laboratory, The First Affiliated Hospital of Medical School of Zhejiang University, Hangzhou, China; 17 Department of Clinical Laboratory, Shandong Provincial Hospital, Jinan, China; 18 Department of Clinical Laboratory, Affiliated Hospital of Inner Mongolia Medical College, Hohhot, China; 19 Department of Clinical Laboratory, Xijing Hospital, Xi’an, China; 20 Department of Clinical Laboratory, Xiangya Hospital, Central South University, Changsha, China; 21 Department of Clinical Laboratory, Beijing Tsinghua Chang Gung Hospital, Beijing, China; 22 Department of Clinical Laboratory, Shanghai Jiaotong University Affiliated Ruijin Hospital, Shanghai, China; 23 Department of Clinical Laboratory, The First Affiliated Hospital of Guangxi Medical University, Nanning, China

**Keywords:** *Streptococcus pneumoniae*, Serotyping, Antimicrobial resistance, Multilocus sequence typing

## Abstract

**Background:**

*Streptococcus pneumoniae*, the leading pathogen of bacterial infections in infants and the elderly, is responsible for pneumococcal diseases with severe morbidity and mortality. Emergence of drug-resistant strains presented new challenges for treatment and prevention. Vaccination has proven to be an effective means of preventing pneumococcal infection worldwide. Detailed epidemiological information of antibiotic susceptibilities and serotype distribution will be of great help to the management of pneumococcal infections.

**Methods:**

A total of 881 *S. pneumoniae* isolates were collected from patients at 23 teaching hospitals in 17 different cities from 2011 to 2016. The main specimen types included sputum, blood, broncho-alveolar lavage fluid, pharyngeal swabs, and cerebrospinal fluid. Minimum inhibitory concentrations (MICs) were determined using the agar dilution method. Capsular serotypes were identified using latex agglutination and quellung reaction test. Molecular epidemiology was investigated using multilocus sequence typing.

**Results:**

*S. pneumoniae* isolates were highly resistant to macrolides, tetracycline, and trimethoprim/sulfamethoxazole. The rate of resistance to penicillin was 51.6% (oral breakpoint). However, levofloxacin and moxifloxacin maintained excellent antimicrobial activity and all of the isolated strains were susceptible to vancomycin.

Twenty-two serotypes were identified among the 881 isolates. Prevalent serotypes were 19F (25.7%), 19A (14.0%), 15 (6.8%), 6B (3.6%), 6A (3.0%), and 17 (2.8%). The overall vaccine coverage rates for 7- and 13-valent pneumococcal conjugate vaccines were 37.5% and 58.3%, respectively. Vaccine coverage rates in young children and economically underdeveloped regions were higher than those in older adults and developed regions. Vaccine-covered serotypes demonstrated higher resistance compared with uncovered serotypes.

Molecular epidemiological typing demonstrated that *S. pneumoniae* showed significant clonal dissemination and that ST271 (120, 28.3%), ST320 (73, 17.2%) and ST81 (27, 6.6%) were the major STs.

**Conclusions:**

High resistance to clinical routine antibiotics was observed for all 881 *S. pneumoniae* strains. Drug resistance varied among different serotypes and age groups. Prevalent serotypes among the isolates were 19F, 19A, 15, 6B, 6A, and 17. Community-acquired strains should also be included in future studies to gain a better understanding of the prevalence and resistance of *S. pneumoniae* in China.

**Electronic supplementary material:**

The online version of this article (doi: 10.1186/s12879-017-2880-0) contains supplementary material, which is available to authorized users.

## Background

*Streptococcus pneumoniae* is a major cause of morbidity and mortality in infants, children, and older adults (≥ 65 years), causing pneumonia and invasive pneumococcal diseases (IPDs) worldwide [1–3]. The disease burden is rapidly worsening in many countries due to the increasing number of people affected by chronic diseases and the increasing disease risks in older age groups [4, 5]. Although IPD is the most severe form with the highest case fatality rate, non-bacteremic pneumonia is the most common manifestation of pneumococcal disease in adults [5]. The population of China is the largest in the world and, given its economic development and improved medical conditions in recent decades, the aging Chinese population is growing; therefore the prevention and control of pneumococcal infections has become an important public health challenge.

Antibiotics are the primary choice for treatment of pneumococcal infections. However, increasing resistance of pneumococci to conventional antibiotics has made the role of antibiotics in the treatment of infection more and more limited [6]. Vaccination has proven to be an effective means of preventing pneumococcal infection worldwide. Immunization with 7-valent pneumococcal conjunctive vaccine (PCV7) and 23-valent pneumococcal polysaccharide vaccine (PPV23) has been recommended for children younger than 24 months of age, high-risk groups, and older adults. For children, PCV7 became commercially available in China in 2008 and was replaced by the 13-valent pneumococcal conjugate vaccine (PCV13) in 2017. Currently, PCV7 is not included in the national immunization program and PCV7 immunization is given only on an individual basis.

A previous study showed that serotype distribution varies with geographical location and age [7]. To effectively control pneumococcal disease in China, the serotype combinations included in vaccines must closely match the distribution of pneumococcal serotypes. Therefore, it is necessary to determine the epidemiology of *S. pneumoniae* after vaccination with the PCV7 available in China. This study analyzed the antimicrobial resistance, serotype distribution, and molecular epidemiology characteristics of 881 pneumococcal isolates from multiple hospitals in China from 2011 to 2016.

## Methods

### Bacterial isolates

This study was conducted at the Peking University People’s Hospital, a teaching hospital located in Beijing, China. A total of 881 *S. pneumoniae* isolates were collected from pediatric and adult patients with pneumococcal infections in 17 cities across China during the years 2011, 2012, 2013, and 2016. The study protocols were approved by Ethics Committee of the Pecking University People’s Hospital and all participants provided written informed consent prior to study commencement. For participants younger than 18 years of age, written informed consent was obtained from each participant’s parents or legal guardian. One isolate was collected from each patient. Duplicate isolates and patients colonized by bacteria but without any clinical evidence of infection were excluded. Isolates were obtained from sputum, blood, broncho-alveolar lavage fluid (BALF), cerebrospinal fluid (CSF), pharyngeal swabs, nasal swabs, and middle ear fluid. Sputum samples from children < 2 years old were collected by nasotracheal aspiration. *S. pneumoniae* isolates from sputums were included if they met the following criteria: less than 10 squamous epithelial cells and more than 25 leukocytes per low power field. Isolates were transported to Peking University People’s Hospital for antibiotic susceptibility testing and serotyping annually. Isolates were identified based on typical colony morphology, Gram staining, optochin sensitivity tests (Oxoid, Hampshire, UK), and Omni serum assays (Statens Serum Institut, Copenhagen, Denmark).

### In vitro antimicrobial susceptibility testing

The agar dilution method was used to determine the minimum inhibitory concentrations (MICs) of the 881 *S. pneumoniae* isolates against 15 antibiotics (penicillin, amoxicillin/clavulanic acid, ceftriaxone, cefuroxime, cefaclor, vancomycin, erythromycin, azithromycin, clarithromycin, tetracycline, levofloxacin, moxifloxacin, trimethoprim/sulfamethoxazole, chloramphenicol and clindamycin) in accordance with the guidelines established by the Clinical and Laboratory Standards Institute (CLSI) [8]. The CLSI 2013 criteria for MICs were applied to classify isolates as susceptible, intermediate, or resistant. The oral penicillin breakpoint was used to classify isolates as penicillin-susceptible (MIC ≤ 0.06 μg/ml), penicillin-intermediate (MIC between 0.12 and 1 μg/ml), or penicillin-resistant (MIC ≥ 2 μg/ml). For ceftriaxone, the non-meningitis breakpoint was used to classify isolates as susceptible (MIC ≤ 1 μg/ml) or resistant (MIC ≥ 4 μg/ml) [9]. *S. pneumoniae* ATCC 49619 was used as the quality control strain and was included in each set of tests to ensure accurate results. MICs were calculated as the MIC_50_ and MIC_90_ (MICs that inhibit 50% and 90% of the isolates, respectively).

### Pneumococcal serotyping

Pneumococcal serotypes/groups were determined for the 881 isolates with Pneumotest-Latex kit (Statens Serum Institut, Copenhagen, Denmark) and type-specific antisera (Statens Serum Institut, Copenhagen, Denmark). The Pneumotest-Latex kit consisted of the 14 latex reagents pools A-I and P-Q. By testing all 14 pools and using the chessboard identification system, it was possible to identify the 23 vaccine serotypes to type/group level. Traditional quellung reaction with type-specific antisera was used for full serotyping of serogroup 19, 6 and 23. Isolates that reacted with the Pneumotest-Latex kit but did not belonged to serotypes or groups included in the PPV23 were classified as Non-vaccine types (NVT). The potential PCV7 and PCV13 vaccine coverage was estimated by calculating the percentage of isolates that expressed the serotypes included in the vaccines and related serotypes (7 for 7F, 9 for 9 V, and 18 for 18C).

### Multilocus sequence typing (MLST) procedure

Multilocus sequence typing (MLST) analysis was performed according to the *S. pneumoniae* MLST protocol [10]. Internal fragments of approximately 550–600 bp from the *aroE*, *gdh*, *gki*, *recP*, *spi*, *xpt*, and *ddl* genes were amplified by polymerase chain reaction using primers described previously [11]. Alleles and sequence types (STs) were assigned using software available at the *Streptococcus pneumoniae* MLST Database web page (http://pubmlst.org/spneumoniae). Analysis of STs and assignment to clonal complexes were performed using all STs found in the online database using the eBURST program. STs were grouped into clonal complexes by their similarity to a central allelic profile. Visualization of phylogenetic results was performed using the PHYLOViZ online tool (http://www.phyloviz.net/).

### Statistical analysis

Data from the antibiotic susceptibility tests were analyzed using WHONET 5.6 software, Windows-based database software developed by the World Health Organization for the management and analysis of microbiological laboratory data with a special focus on the analysis of antimicrobial susceptibility test results. χ^2^ and Fisher’s exact probability tests were performed using SPSS for Windows (version 18.0; SPSS, Chicago, IL, USA) to compare proportions. *P* values < 0.05 were considered statistically significant.

## Results

### Profile of pneumococcal isolates

During the study period, a total of 881 non-duplicated isolates were collected from 23 teaching hospitals in 17 cities. The number of isolates varied with years and regions and it was detailed in the Additional file 1: Table S1. Of the 881 *S. pneumoniae* isolates, 131 were obtained from pediatric patients aged 0 to 2 years (≤ 2 years old), 45 were obtained from pediatric patients aged 2 to 5 years (> 2 but ≤ 5 years old), 43 were obtained from patients aged 5 to 18 years (> 5 but ≤ 18 years old), 408 were obtained from adults aged 18 to 65 years (> 18 but ≤ 65 years old), and 254 were obtained from patients > 65 years old. Sputum was the most common specimen source, accounting for 73.1% of samples (644 strains), followed by blood (56 strains), BALF (56 strains), pharyngeal swabs (22 strains) and CSF (13 strains) Table 1. Strains isolated from sterile sites, such as blood, CSF, and pleural fluid, were classified as IPD strains. The 17 cities were divided into three groups based on the per capita gross domestic product (GDP) of the province in which the city is located (China Statistical Yearbook 2016, http://www.stats.gov.cn/tjsj/ndsj/2016/indexeh.htm). Cities in group 1 had a per capita GDP of more than 60,000 Chinese yuan (CNY) and included Tianjin, Beijing, Shanghai, Jiangsu, Zhejiang, Inner Mongolia, Guangdong, and Shandong. Cities in group 2 had a per capita GDP between 50,000 and 60,000 CNY and included Chongqing, Hubei, Jilin, Shaanxi, and Liaoning. Cities in group 3 had a per capita GDP of less than 50,000 CNY and included Ningxia, Hunan, Xinjiang, and Guangxi.

**Table 1:** Distribution of *Streptococcus pneumoniae* specimens by age group and year collected

Specimen type	Number	%	Age groups					Years			
			≤ 2 years	> 2 and ≤ 5 years	> 5 and ≤ 18 years	> 18 and ≤ 65 years	> 65 years	2011	2012	2013	2016
Sputum	644	73.1	121	33	26	259	205	34	210	166	234
Blood	56	6.4	3	3	3	35	12	3	18	17	18
Broncho-alveolar lavage	56	6.4			1	37	18		13	17	26
Pharyngeal swabs	22	2.5	3	6	4	8	1	3	8	4	7
Cerebrospinal fluid	13	1.5			1	9	3		2	4	7
Eye secretion	10	1.1		2	2	5	1	1	1	4	4
Secretion	9	1.0	1		1	5	2		5		4
Pus	8	0.9	1			6	1		4	4	
Nasal swabs	6	0.7	2			4			3	3	
Wound swab	6	0.7				5	1		2	3	1
Pleural fluid	6	0.7				5	1		2		4
Tissue	6	0.7				2	4			5	1
Sinus swab	4	0.5			2	2					4
Tracheal	4	0.5				3	1			4	
Middle ear fluid	3	0.3			1	1	1		1	2	
Abdominal fluid	3	0.3				3				1	2
Bile	2	0.2					2		1		1
Urine	2	0.2				2				1	1
Bone marrow	1	0.1				1				1	
Vaginal swab	1	0.1				1				1	
Not specified	19	2.2		1	2	15	1				19

### Antimicrobial susceptibility of *Streptococcus pneumoniae*

**Fig. 1:**
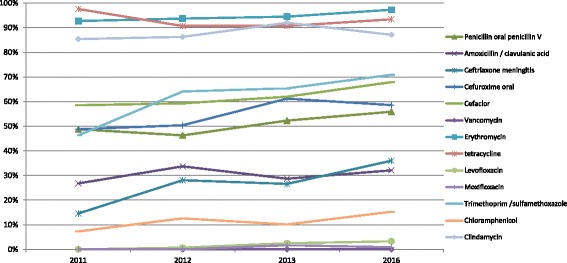
Changes in resistance of *S. pneumoniae* to different antimicrobial agents from 2011 to 2016

The in vitro activities of the tested antimicrobial agents against the 881 *S. pneumoniae* strains are shown in Table 2. Based on the MIC breakpoints of oral penicillin, the percentages of penicillin-resistant *S. pneumoniae* (PRSP), penicillin-intermediate *S. pneumoniae* (PISP), and penicillin-susceptible *S. pneumoniae* (PSSP) isolates were 51.6% (455/881), 12.3% (108/881), and 36.1% (318/881), respectively. The rates of resistance of *S. pneumoniae* to erythromycin, azithromycin, and clarithromycin were 95.2%, 96.9%, and 92.5%, respectively. Macrolides exhibited weak activity against all of the isolates. Of the 839 strains (95.2%) that were resistant to erythromycin, 776 (92.5% of all strains) were also resistant to clindamycin. *S. pneumoniae* susceptibilities to amoxicillin/clavulanic acid and ceftriaxone were comparable (using non-meningitis breakpoints), but the rate of resistance to amoxicillin/clavulanic acid was higher than to ceftriaxone (using non-meningitis breakpoints). Overall, the resistance of *S. pneumoniae* to penicillin (based on breakpoints of oral penicillin) increased from 48.8% in 2011 to 55.9% in 2016 (*P* = 0.390, Fig. 1). The proportion of PRSP isolates remained stable and there were no statistical differences in rates of PRSP from 2011 to 2016 (Table 3). During the same time period, the proportion of PSSP isolates fell from 39% in 2011 to 33.6% in 2016. The MIC_50_ of penicillin was 1 μg/ml in 2011 and increased to 2 μg/ml in 2016, indicating a trend towards an increase in resistance to penicillin. Trends towards increases in resistance were also observed for other β-lactams and cephalosporins. However, fluoroquinolones maintained excellent activity against *S. pneumoniae*; the MIC_90_ values for levofloxacin and moxifloxacin were 1 μg/ml and 0.25 μg/ml, respectively. There were 19 *S. pneumoniae* strains resistant to levofloxacin and the MIC values were between 8 and 32 μg/ml. All of the strains were susceptible to vancomycin and no vancomycin-resistant strains were found during the study period. The rates of resistance of penicillin non-susceptible *S. pneumoniae* (PNSP) to other antimicrobial agents were also significantly higher than those of PSSP. However, no statistical differences in resistance to azithromycin, clarithromycin, levofloxacin, or moxifloxacin were found between PSSP and PNSP strains. The proportion of PSSP isolates varied with patient age; PSSP was more common among older adults compared with children (42.0% vs 18.7%, *P* < 0.001). There were no significant differences in the proportion of PNSP isolates between IPD and non-IPD strains. The rate of PSSP isolated from patients in emergency units was higher than in other hospital wards (*P* = 0.041). Similarly, the rate of PNSP isolated from patients in intensive care units (ICUs) was higher than in other wards (*P* = 0.017, Table 3).

**Table 2:** In vitro activity of 14 antimicrobial agents against 881 isolates of *Streptococcus pneumoniae*

Antimicrobial agent	PSSP (*n* = 318)				PISP (*n* = 108)				PRSP (*n* = 455)				P
	R%	MIC_50_	MIC_90_	MIC range	R%	MIC_50_	MIC_90_	MIC range	R%	MIC_50_	MIC_90_	MIC range	
Penicillin (oral)	0	0.016	0.032	.004–.064	0	1	1	.125–1	100	4	4	2–64-	–
Amoxicillin/clavulanic acid	0.3	0.016	0.032	.008–32	0	1	2	.008–2	60.7	8	16	.016–128	< 0.00001
Ceftriaxone (non meningitis)	0	0.016	0.064	.016–1	0.9	0.5	1	.016–4	40.7	2	8	.25–512	–
Cefuroxime oral	0.6	0.032	0.125	.016–8	45.4	2	4	.016–32	97.8	16	32	.008–512	< 0.00001
Cefaclor	2.2	1	2	.016–32	88.9	128	256	1–512-	99.8	>256	>256	2–512-	< 0.00001
Vancomycin	0	0.25	0.25	.008–.5	0	0.25	0.5	.064–.5	0	0.25	0.25	.064–1	–
Erythromycin	89	16	>256	.016–512	96.3	32	>256	.032–512	99.3	>256	>256	.032–512	< 0.00001
Azithromycin	94.6	32	>256	.016–512	94.4	32	>256	.032–512	99.3	>256	>256	.032–512	0.135
Clarithromycin	88.3	32	>256	.016–512	80.6	32	>256	.016–512	98.6	>256	>256	.032–512	0.063
Tetracycline	84.6	32	64	.064–128	92.6	16	64	.25–64	97.1	32	64	.25–128	< 0.00001
Levofloxacin	1.3	1	1	.025–32	5.6	1	1	.25–32	2	1	1	.5–64	0.163
Moxifloxacin	0.6	0.125	0.25	.016–16	1.9	0.125	0.25	.016–8	0.7	0.125	0.25	.016–16	0.666
Trimethoprim/sulfamethoxazole	34.9	0.5	8	.032–64	60.2	4	8	.064–32	89.5	8	16	.125–128	< 0.00001
Clindamycin	74.2	128	256	.016–512	88	128	256	.016–256	97.8	256	256	.016–512	< 0.00001

**Table 3:** Proportion of PSSP and PNSP isolates by age, year, and vaccination status

Groups		Number of isolates	PSSP %	PNSP %	*P* value
Age	≤ 2 years	131	19.1	80.9	< 0.00001
	> 2 and ≤ 5 years	45	15.6	84.4	0.003083
	> 5 and ≤ 18 years	43	20.9	79.1	0.032561
	> 18 and ≤ 65 years	408	42.2	57.8	0.000646
	> 65	254	41.7	58.3	0.029919
Years	2011	41	39	61	0.700859
	2012	270	39.3	60.7	0.210449
	2013	237	35.9	64.1	0.897489
	2016	333	33.6	66.4	0.215055
PCV7					< 0.00001
	PCV7 covered	330	12.1	87.9	
PCV13					< 0.00001
	PCV13 covered	514	14.8	85.2	
IPD					0.223755
	IPD	75	42.7	57.3	
Ward types	unknown	25	36	64	0.982413
	emergency	55	49.1	50.9	0.040078
	out patient	103	34	66	0.616566
	in patient	635	36.9	63.1	0.524408
	ICU	63	22.2	77.8	0.016520
Major serotypes	19F	226	3.5	96.5	< 0.00001
	19A	123	5.7	94.3	< 0.00001
	15	58	46.6	53.4	0.089938
	6B	31	19.4	80.6	0.046833
	6A	26	3.8	96.2	0.000491
	17	25	84	16	< 0.00001
	14	21	0	100	–
	23F	19	10.5	89.5	0.018531
	NVT	234	69.2	30.8	< 0.00001

#### Serotyping and vaccine coverage

**Fig. 2:**
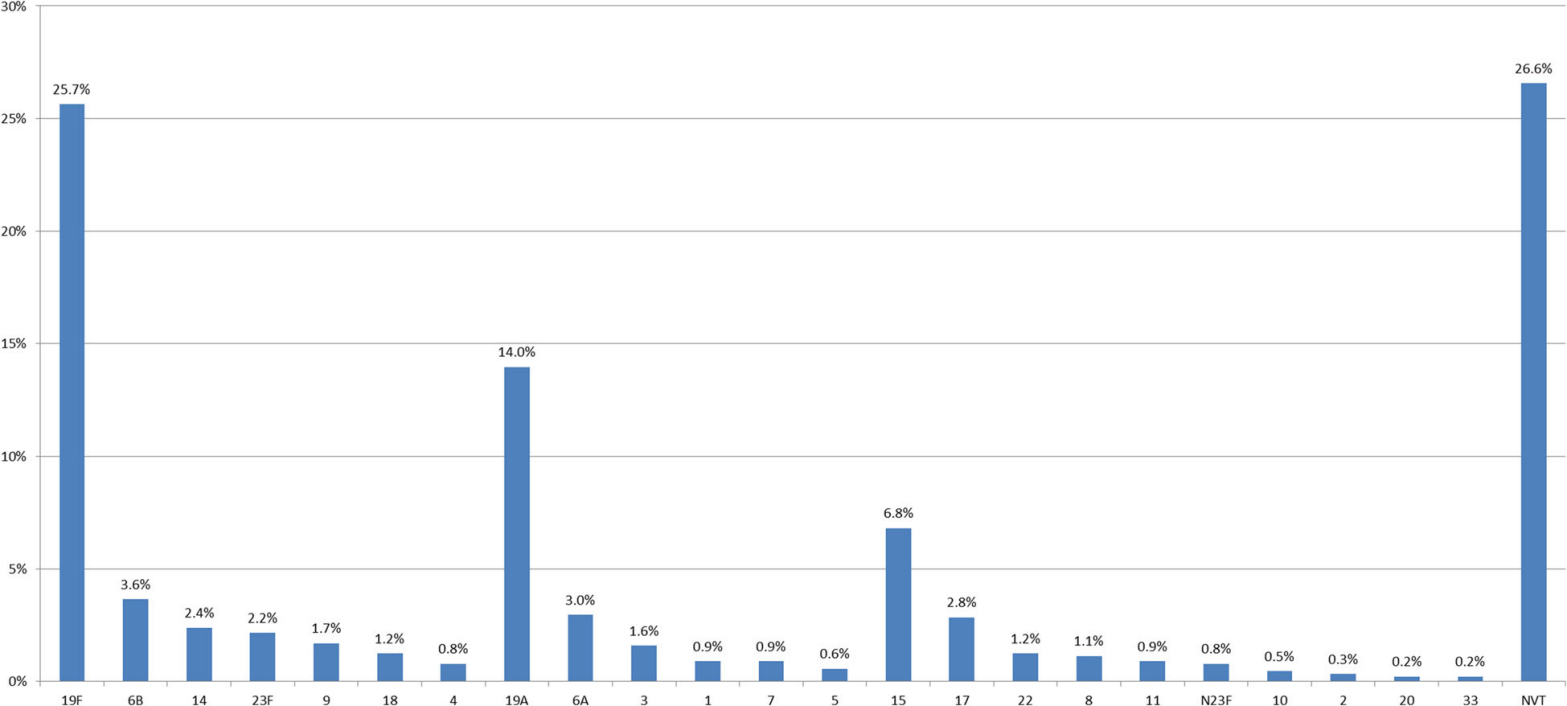
Distribution and proportion of 881 *S. pneumoniae* strains collected between 2011 and 2016. N23F strains belong to serogroup 23 but not serotype 23F. NVT: serotypes not included in the PPV23. Serotypes 4, 6B, 9, 14, 18, 19F, and 23F are included in PCV7. Serotypes 1, 3, 4, 5, 6A, 6B, 7F, 9, 14, 18C, 19A, 19F, and 23F are included in PCV13

Of the 881 isolates, 234 isolates were non-typable and 22 different kinds of serotypes were identified. The serotype distribution of these *S. pneumoniae* isolates is shown in Fig. 2. Among the typable isolates, 19F (25.7%), 19A (14.0%), 15 (6.8%), 6B (3.6%), 6A (3.0%), and 17 (2.8%) were the most common serotypes. The most common serotypes in all age groups were 19F and 19A.

The overall vaccine coverage rates for PCV7 and PCV13, which include isolates 1, 3, 5, 6A, 7, and 19A, were 37.5% and 58.3%, respectively. Importantly, vaccine coverage rates varied by patient age; vaccine coverage rates in young children were significantly higher than in older adults (Table 4).

**Table 4:** Coverage (%) of PCV7 and PCV13 in different age groups from 2011 to 2016

Age groups	PCV7 covered				PCV13 covered			
	2011	2012	2013	2016	2011	2012	2013	2016
≤ 2 years	42.9	48.1	37.2	55.6	71.4	74.1	62.8	59.3
> 2 and ≤ 18 years	50	52.6	41.7	37.8	62.5	84.2	70.8	64.9
> 18 and ≤ 65 years	43.8	31.5	36.7	31.8	62.5	56.5	52	51.2
> 65 years	30	41.1	38.9	35.4	40	58.9	63.9	53.5

Immunization coverage in this study varied considerably by geographic area. The highest coverage of PCV7 and PCV13 was in Jiangsu with rates of 63.2% and 78.9%, respectively. In contrast, the lowest coverage was in Jilin with rates of 12.9% and 32.3%, respectively. We also found that vaccine coverage was associated with economic development. There was no difference in PCV7 or PCV13 coverage between groups 1 and 2, whereas PCV7 and PCV13 coverage was significantly higher in group 3 compared with groups 1 and 2 (Table 5).

**Table 5:** Coverage (%) of PCV7 and PCV13 in different regions in China

Group by GDP	Province	Number	non-PCV7 covered (%)	PCV7 covered (%)	*P* value	non-PCV13 covered (%)	PCV13 covered (%)	*P* value
Group 1	Beijing	175	120 (68.6)	55 (31.4)		84 (48.0)	91 (52.0)	
Group 1	Guangzhou	91	50 (54.9)	41 (45.1)		25 (27.5)	66 (72.5)	
Group 1	Zhejiang	82	61 (74.4)	21 (25.6)		44 (53.7)	38 (46.3)	
Group 1	Jiangsu	38	14 (36.8)	24 (63.2)		8 (21.1)	30 (78.9)	
Group 1	Inner Mongolia	19	11 (57.9)	8 (42.1)		11 (57.9)	8 (42.1)	
Group 1	Shandong	29	20 (69.0)	9 (31.0)		13 (44.8)	16 (55.2)	
Group 1	Shanghai	59	34 (57.6)	25 (42.4)		24 (40.7)	35 (59.3)	
Group 1	Tianjin	50	33 (66.0)	17 (34.0)		29 (58.0)	21 (42.0)	
Group 1		543	343 (63.2)	200 (36.8)	0.627101	238 (43.8)	305 (56.2)	0.097211
Group 2	Chongqing	79	44 (55.7)	35 (44.3)		30 (38.0)	49 (62.0)	
Group 2	Jilin	31	27 (87.1)	4 (12.9)		21 (67.7)	10 (32.3)	
Group 2	Liaoning	44	37 (84.1)	7 (15.9)		20 (45.5)	24 (54.5)	
Group 2	Hubei	52	31 (59.6)	21 (40.4)		22 (42.3)	30 (57.7)	
Group 2	Shaanxi	31	15 (48.4)	16 (51.6)		7 (22.6)	24 (77.4)	
Group 2		237	154 (65.0)	83 (35.0)	0.364746	100 (42.2)	137 (57.8)	0.844537
Group 3	Hunan	11	5 (45.5)	6 (54.5)		3 (27.3)	8 (72.7)	
Group 3	Guangxi	7	5 (71.4)	2 (28.6)		3 (42.9)	4 (57.1)	
Group 3	Ningxia	39	19 (48.7)	20 (51.3)		10 (25.6)	29 (74.4)	
Group 3	Xinjiang	44	25 (56.8)	19 (43.2)		13 (29.5)	31 (70.5)	
Group 3		101	54 (53.5)	47 (46.5)	0.045169	29 (28.7)	72 (71.3)	0.005041

The sensitivities of the *S. pneumoniae* serotypes to antimicrobial agents varied significantly. In serogroups 19 (including 19F and 19A), 6 (including 6A and 6B), 14, and 23F, the proportion of PNSP isolates was significantly higher compared with the other common serotypes (Table 3), whereas the proportion of PSSP isolates was higher in serotypes 15 and 17 and in the non-typable strains. The proportion of PNSP isolates was higher among vaccine-covered strains (87.9% for PCV7 and 85.2% for PCV13) than non-covered strains (49.4% for PCV7 and 33.8% for PCV13).

#### Multilocus sequence typing

**Fig. 3:**
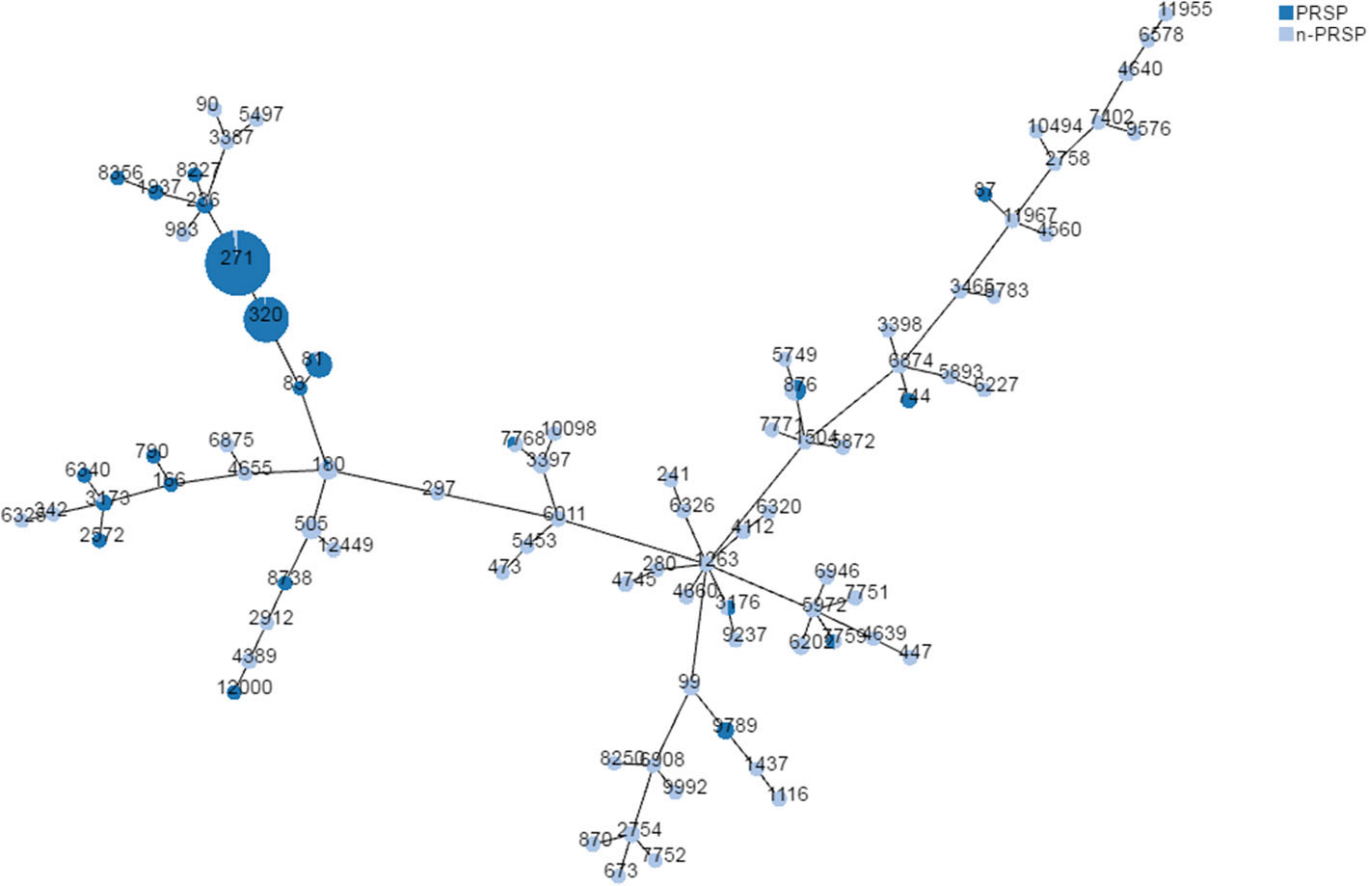
Phylogenetic tree of 424 S. pneumoniae strains generated by the PHYLOViZ online tool

Of the 881 strains, 424 (strains isolated in 2016 were not tested by MLST) were identified as 82 STs. Among the tested strains, ST271 (120, 28.3%), ST320 (73, 17.2%), and ST81 (27, 6.6%) were the most common STs. Of the 19F isolates that were identified with specific STs, 98 (77.8%) were ST271 and 15 (11.9%) were ST320. Of the 19A isolates, 55 (75.3%) were ST320 and 15 (20.6%) were ST271. We found that 19F–ST271 and 19A–ST320 isolates were more resistant to several of the tested antibiotics, especially β-lactams (Table 6). The phylogenetic tree generated using the PHYLOViZ online tool showed that all of the tested strains exhibited obvious clonal aggregation, with the vast majority of the population made up of resistant clones and serotypes, whereas susceptible clones and serotypes showed a decentralized pattern (Fig. 3).

**Table 6:** Sequence types, serotypes, antibiotic resistance rates (%), and age distributions for 424 *Streptococcus pneumoniae* isolates analyzed by MLST

Clonal Complex	ST	Number	Serotypes (Number)	Resistance rates of different antibiotics			Number of strains in different ages				
				Penicillin	Levofloxacin	Erythromycin	<= 2	(2,5]	(5,18]	(18,65]	> 65
CC271	271	120	19F(98), 19A(15), NVT(5), 15(1), 1(1)	97.5	0	100	35	7	9	41	28
	320	73	19A(55), 19F(15), 18(1), 15(1), NVT(1)	98.6	2.7	100	12	10	0	29	22
	236	6	19F(5), NVT(1)	83.3	16.7	100	1	0	0	3	2
	1937	2	19F(2)	100	0	100	0	0	0	1	1
CC81	81	28	23F(8), 15(6), NVT(6), N23F(3), 6B(2), 3(1), 17(1), 18(1)	92.9	3.6	100	5	3	0	15	5
	83	1	19F(1)	100	0	100	1	0	0	0	0
CC876	876	17	14(11), NVT(3), 18(2), 4(1)	47.1	5.9	100	2	3	0	10	2
	5749	1	14(1)	0	0	100	0	0	0	0	1
CC505	505	14	NVT(9), 3(3), 17(1), 19F(1)	0	0	92.9	0	0	2	6	6
	12,449	2	NVT(2)	0	0	100	0	0	0	2	0
CC180	180	13	NVT(12), 8(1)	7.7	0	92.3	1	0	0	8	4
	297	1	NVT(1)	0	0	100	0	0	0	0	1
CC3397	3397	9	15(7), 8(2)	0	0	88.9	3	1	0	3	2
	7768	3	15(3)	33.3	0	100	2	1	0	0	0
	10,098	1	15(1)	0	0	100	1	0	0	0	0
CC1263	6011	4	15(2), 4(1), NVT(1)	0	0	100	0	0	0	2	2
	4660	2	9(1), NVT(1)	0	0	100	0	0	0	2	0
	1263	2	9(1), NVT(1)	0	0	100	0	0	0	1	1
	280	1	1(1)				0	0	0	1	0
CC5972	6946	4	17(3), 8(1)	0	25	100	0	0	0	2	2
	5972	3	NVT(2), 5(1)	0	0	100	0	0	0	2	1
	7759	2	3(1), NVT(1)	50	0	100	0	0	0	2	0
CC2754	2754	6	NVT(3), 15(2), 8(1)	0	0	100	0	0	0	5	1
	7752	1	NVT(1)	0	0	100	0	0	0	0	1
CC3173	3173	5	6A(4), 6B(1)	80	0	100	4	0	0	1	0
	6340	1	19F(1)	100	0	100	0	0	0	0	1
CC2758	2758	1	7(1)	0	0	100	1	0	0	0	0
	7402	1	11(1)	0	0	100	0	0	0	0	1
	11,967	1	NVT(1)	0	0	100	0	0	0	0	1
CC3387	3387	2	NVT(2)	0	0	100	2	0	0	0	0
	90	1	6B(1)	0	0	100	1	0	0	0	0
CC2912	2912	1	6B(1)	0	0	0	0	0	0	0	1
	8738	1	NVT(1)	100	0	100	1	0	0	0	0
CC342	342	1	17(1)	0	0	100	0	0	0	0	1
	6325	1	1(1)	0	0	100	0	0	0	1	0
Singletons	9789	7	6B(3), 6A(2), NVT(2)	100	0	100	2	0	0	4	1
	6202	7	15(6), 10(1)	0	0	100	0	0	0	7	0
	99	5	1(1),3(1),4(1),5(1),19F(1)	0	0	100	0	0	0	2	3
	4389	4	NVT(4)	0	0	50	0	0	0	1	3
	OTHER	69	NVT(22), 17(7), 18(6), 15(4), 19A(3), 1(2), 14(2), 19F(2), 2(2), 20(2), 22(2), 23F(2), 3(2), 7(2), 8(2), 9(2), N23F(2), 4(1), 6A(1), 6B(1)	14.5	0	89.9	16	3	3	30	17

## Discussion

Infections caused by *S. pneumoniae* have traditionally been treated with β-lactams, to which this species was extremely sensitive when penicillin was first introduced in the 1940s [12]. However, resistance was first observed in the 1960s and has continued to increase throughout the world in recent decades [12, 13]. The emergence of resistance to penicillin and other β-lactam antibiotics in pneumococci has led to the increased adoption of macrolides, fluoroquinolones, and other non-β-lactam antibiotics to treat pneumococcal infections [14]. However, resistance to antimicrobials continues to increase, complicating efforts to treat pneumococcal disease in both adults and children.

The data from this study showed that *S. pneumoniae* isolated from adults and children during the investigation period were highly resistant to β-lactams, macrolides, and trimethoprim/sulfamethoxazole, which is consistent with a previous study [15]. Based on the MIC breakpoints of oral penicillin, the proportion of PRSP increased from 48.8% in 2011 to 55.9% in 2016. MICs of most of the antimicrobial agents tested against PRSP were higher than against PSSP, indicating co-resistance between these antimicrobials. Consequently, the increasing prevalence of multidrug resistant (MDR) in *S. pneumoniae* in China is becoming a serious health threat.

Macrolides, including erythromycin, azithromycin, and clarithromycin, had the lowest antibacterial activity against both PNSP and PSSP strains, with MIC_90_ values greater than 256 μg/ml. Since the early 1990s, the American Thoracic Society treatment guidelines have listed macrolide antibiotics as the first-line empiric therapy for outpatients with community-acquired pneumonia [16], resulting in the widespread use of these agents. In Europe, resistance of *S. pneumoniae* to erythromycin has been reported to be between 14.7 and 17.1% [17]. However, more than 90% of clinical *S. pneumoniae* strains in this study were found to be resistant to macrolides; therefore macrolides should be used cautiously as empiric therapy against pneumococcal infection in China. Abuse of macrolides in outpatient practices and the clonal spread of MDR strains is likely responsible for the high prevalence of macrolide resistance in China [18]. Additionally, macrolide-resistant *S. pneumoniae* strains identified in this study were found to be co-resistant to other antibacterials, such as tetracycline (94.2%), clindamycin (92.5%), and penicillin (53.9%; oral penicillin V). Other researchers have shown that the majority of MDR *S. pneumoniae* in China are macrolide-lincosamide-streptogramin B resistant and that they carry the *erm(B)* gene [19]. Multiple antibiotic resistance is widespread in China and an increase in MDR *S. pneumoniae* strains has also been observed in other parts of Asia such as Japan, Korea, and Taiwan [20]. In contrast, fluoroquinolones showed exceptional activity against *S. pneumoniae* in this study, which is in accordance with other reports [18, 21, 22], indicating that fluoroquinolones could be a better option for the treatment of pneumococcal infections in adult patients.

Interestingly, in this study, strains isolated from patients in emergency units were more susceptible to penicillin, whereas strains isolated from patients in ICUs were more resistant to penicillin. This could indicate that *S. pneumoniae* strains within the community were more susceptible to penicillin, whereas more resistant strains, for which treatments were more likely to fail, developed within medical institutions.

In this study, the proportion of PNSP isolates in serotypes 19F, 19A, and 6B was higher than in other serotypes. Previous studies have indicated that recombination efficiency varies with *S. pneumoniae* serotype, with certain strains having been identified as particularly efficient at recombination. For example, serotypes 3 and 18C were found to be much less transformable with a selective marker compared with 19F, 19A, 23F, 6B, and 14 [23]. Interspecies and intraspecies genetic transformations likely play an important role in most multi-antimicrobial resistance mechanisms in pneumococci [24].

Vaccination is an alternative method of preventing pneumococcal infection. It has been reported that the prevalence of vaccine-covered serotypes decreased significantly after large-scale application of PCV7. Studies have shown that the introduction of pneumococcal conjugate vaccines has not only reduced the burden of pneumococcal disease in children [25], but has also greatly impacted the burden of disease in adults by preventing the spread of vaccine-related resistant strains to adults [26, 27]. PCV7, which was replaced by PCV13 in 2016, has been used in China since 2008 on an individual basis. In this study, the average vaccine coverage of PCV7 (37.5%) and PCV13 (58.3%) in the population was similar to the vaccine coverage before they were introduced into China [28], suggesting that the effects of PCV7 and PCV13 in China were limited. Exclusion from the national immunization program and high prices could be responsible for low vaccination rates and poor herd immunity in the target population [29]. We also found that in areas with better economic development, vaccine coverage was lower than in less economically developed regions. This is likely due to historically higher rates of vaccine use in economically developed regions, resulting in greater herd protection and a subsequent decline in the rate of vaccine coverage. Considering the protective effect of vaccines reported in other countries and the high coverage of PCV13 in both children and adults in China, inclusion of PCV13 in the national immunization program could result in significant changes in the serotype distribution of *S. pneumoniae* in both children and adults.

There were some limitations to this study. Most importantly, all strains were obtained from sophisticated medical institutions, whereas no strains were obtained from community-based clinics. Patients are typically admitted to large medical institutions when empirical treatment has failed or when existing diseases worsen. Patients infected with strains that have lower levels of resistance and virulence are more likely to be cured by the empirical treatment regimen and would not need to go to a large hospital for further medication. Because of this screening mechanism, the data from this study only explain resistance and serotype distribution status of *S. pneumoniae* isolated from hospitals. Additionally, while the effect of PCV13 has been evaluated mainly in IPD strains [30], the number of IPD strains in this study was very limited. This may be due to less blood cultures were prescribed in China. The proportion of blood cultures in all microbiology specimens varied with hospitals in this study, ranging from 20% to 50%. The percentages of blood-cultures were higher in large cities and teaching hospitals and were lower in small cities and primary hospitals. In China, doctors diagnose respiratory infections relied a lot on X-ray imaging findings and white blood cell count, both of them returned results on the same day. Although doctors also prescribed microbiology cultures (usually sputum culture) pathogen detection, doctors may empirically use antibiotics if X-ray imaging and white blood cell count indicating a bacterial infection. Further study on IPD strains is required to assess the effectiveness of the PCV13 vaccine in China. The number of strains varied with years and regions, and the number of strains in some years and regions was relatively small. A very small sample size would inevitably introduce random errors, especially in the comparison of vaccine coverage rates between different age groups and regions. Therefore, in order to clarify the resistance and serotype distribution of *S. pneumoniae* in the population, our future studies will assess strains from primary health care institutions in the community.

## Conclusions

High resistance to β-lactams and macrolides was observed of all 881 *S. pneumoniae* strains. However, fluoroquinolones maintained excellent activity against *S. pneumoniae*. Drug resistance varied among different serotypes and age groups. Serotypes 19F, 19A, 15, 6B, 6A and 17 were the most common serotypes. The serotype 19F, 19A, 6A, 6B, 14 and 23F demonstrated higher resistance compared with other serotypes. Vaccine coverage in this study varied considerably associated geographic area and economic development. Through molecular biology analysis, obvious clonal aggregation was observed. Community-acquired and more invasive strains should also be included in future studies to gain a better understanding of the prevalence and resistance of *S. pneumoniae* in China.

## Additional file

Additional file 1: Table S1. Number of *Streptococcus pneumoniae* strains in different cities and years. This table describes the numbers of strains in different cities and years. The cities in this table were divided into 3 group based on the annual per capita GDP. (DOCX 16 kb)

## Abbreviations

ATCC: American Type Culture Collection
BALF: Broncho-alveolar lavage fluid
CLSI: Clinical and Laboratory Standards Institute
CNY: Chinese yuan
CSF: Cerebrospinal fluid
GDP: Gross domestic product
ICU: Intensive care unit
IPD: Invasive pneumococcal disease
MDR: Multidrug resistant
MIC: Minimum inhibitory concentration
MLST: Multilocus sequence typing
PCV13: 13-valent pneumococcal conjugate vaccine
PCV7: 7-valent pneumococcal conjunctive vaccine
PISP: Penicillin-intermediate *S. pneumoniae*
PNSP: Penicillin non-susceptible *S. pneumoniae*
PPV23: 23-valent pneumococcal polysaccharide vaccine
PRSP: Penicillin-resistant *S. pneumoniae*
PSSP: Penicillin-susceptible *S. pneumoniae*
ST: Sequence type
SXT: Trimethoprim/sulfamethoxazole
