# Supplementary material for: Serotype distribution and antibiotic resistance of Streptococcus pneumoniae isolates from 17 Chinese cities from 2011 to 2016
Source: BMC Infect Dis. 2017 Dec 29;17:804. doi: 10.1186/s12879-017-2880-0 (PMC5747162; doi:10.1186/s12879-017-2880-0)
Supplement: Additional file 1: Table S1. — Number of Streptococcus pneumoniae strains in different cities and years. This table describes the numbers of strains in different cities and years. The cities in this table were divided into 3 group based on the annual per capita GDP. (DOCX 16 kb) [file 12879_2017_2880_MOESM1_ESM.docx]

Table S1. Number of *Streptococcus pneumoniae* strains in different cities and years.

| Group of GPD | Name of province | | Name of Cities | | Total | | Years | | | | | | | |
| --- | --- | --- | --- | --- | --- | --- | --- | --- | --- | --- | --- | --- | --- | --- |
|  |  |  |  |  |  |  | 2011 | | 2012 | | 2013 | | 2016 | |
| Group 1 | Beijing | | Beijing | | 175 | |  | | 63 | | 55 | | 57 | |
| Group 1 | Guangdong | | Guangzhou | | 91 | |  | | 40 | | 38 | | 13 | |
| Group 1 | Zhejiang | | Hangzhou | | 82 | |  | | 44 | | 24 | | 14 | |
| Group 1 | Shanghai | | Shanghai | | 59 | |  | | 19 | | 17 | | 23 | |
| Group 1 | Tianjin | | Tianjin | | 50 | | 2 | | 16 | | 19 | | 13 | |
| Group 1 | Jiangsu | | Nanjing | | 38 | | 7 | | 11 | | 20 | |  | |
| Group 1 | Shandong | | Jinan | | 29 | |  | |  | |  | | 29 | |
| Group 1 | Inner Mongolia | | Hohhot | | 19 | |  | |  | |  | | 19 | |
| Group 2 | Chongqing | | Chongqing | | 79 | | 32 | | 21 | | 24 | | 2 | |
| Group 2 | Hubei | | Wuhan | | 52 | |  | | 17 | | 13 | | 22 | |
| Group 2 | Liaoning | | Shenyang | | 44 | |  | | 20 | | 15 | | 9 | |
| Group 2 | Shaanxi | | Xi'an | | 31 | |  | | 19 | | 12 | |  | |
| Group 2 | Jilin | | Changchun | | 31 | |  | |  | |  | | 31 | |
| Group 3 | Xinjiang | | Urumqi | | 44 | |  | |  | |  | | 44 | |
| Group 3 | Ningxia | | Yinchuan | | 39 | |  | |  | |  | | 39 | |
| Group 3 | Hunan | | Changsha | | 11 | |  | |  | |  | | 11 | |
| Group 3 | | Guangxi | | Nanning | | 7 | |  | |  | |  | | 7 |
